# The Association of Thyroid Hormone Changes with Inflammatory Status and Prognosis in COVID-19

**DOI:** 10.1155/2021/2395212

**Published:** 2021-08-13

**Authors:** Ceyda Dincer Yazan, Can Ilgin, Onur Elbasan, Tugce Apaydin, Saida Dashdamirova, Tayfun Yigit, Uluhan Sili, Aysegul Karahasan Yagci, Onder Sirikci, Goncagul Haklar, Hulya Gozu

**Affiliations:** ^1^Marmara University School of Medicine, Department of Endocrinology and Metabolism, Istanbul, Turkey; ^2^Marmara University School of Medicine, Department of Public Health, Istanbul, Turkey; ^3^Marmara University School of Medicine, Department of Biochemistry, Istanbul, Turkey; ^4^Marmara University School of Medicine, Department of Infectious Diseases and Clinical Microbiology, Istanbul, Turkey; ^5^Marmara University School of Medicine, Department of Microbiology, Istanbul, Turkey

## Abstract

**Background:**

COVID-19 infection may have multiorgan effects in addition to effects on the lungs and immune system. Recently, studies have found thyroid function abnormalities in COVID-19 cases which were interpreted as euthyroid sick syndrome (ESS) or destructive thyroiditis. Therefore, in this study, we aimed to evaluate the thyroid function status and thyroid autoimmunity in COVID-19 patients. *Material and Method*. 205 patients were included. The medical history and laboratory parameters at admission were collected from medical records. Serum thyroid-stimulating hormone (TSH), free thyroxine (FT4), free triiodothyronine (FT3), thyroid peroxidase antibody, and thyroglobulin antibody were measured, and patients were classified according to thyroid function status.

**Results:**

34.1% of the patients were euthyroid. Length of hospitalization (*p* < 0.001), rate of oxygen demand (*p* < 0.001), and intensive care unit (ICU) admission (*p*=0.022) were lower, and none of the euthyroid patients died. 108 (52.6%) patients were classified to have ESS, 57 were classified as mild, and 51 were moderate. The inflammatory parameters were higher in patients with moderate ESS. In cluster analysis, a high-risk group with a lower median FT3 value (median = 2.34 ng/L; IQR = 0.86), a higher median FT4 value (median = 1.04 ng/dL; IQR = 0.33), and a lower median TSH value (median = 0.62 mIU/L; IQR = 0.59) included 8 of 9 died patients and 25 of the 31 patients that were admitted to ICU. *Discussion*. Length of hospitalization, oxygen demand, ICU admission, and mortality were lower in euthyroid patients. Moreover, none of the euthyroid patients died. In conclusion, evaluation of thyroid function tests during COVID-19 infection may give information about the prognosis of disease.

## 1. Introduction

COVID-19 affects not only lungs but also vascular endothelial cells, heart, brain, kidneys, intestine, liver, pharynx, and others including the thyroid gland [1, 2].

Preliminary studies on COVID-19 have shown thyroid function abnormalities that were interpreted as a euthyroid sick syndrome (ESS) [3–13] (Table 1) or thyrotoxicosis associated with destructive thyroiditis [5, 6]. However, several limitations such as small study population, incomplete evaluation of thyroid function tests, no control group, and drug interference exist in those studies [3–13].

Therefore, we aimed to make a descriptive study to evaluate COVID-19, especially its severity, in relation to thyroid function tests and thyroid autoimmune parameters in a large patient population.

## 2. Material and Method

The study was approved by the Local Ethics Committee of Marmara University School of Medicine (11.05.2020/ethics no.: 535) and Turkish Ministry of Health.

Two hundred and five patients with reverse-transcription polymerase chain reaction- (RT-PCR-) confirmed COVID-19 who were admitted to Marmara University Education and Research (E&R) Hospital between April and October 2020 were enrolled. None of them have had previous thyroid disease, used any thyroid medications or glucocorticoids, or had pregnancy. All patients were evaluated for thyroid-stimulating hormone (TSH), free triiodothyronine (FT3), free thyroxine (FT4), thyroglobulin antibody (TGAb), and thyroid peroxidase antibody (TPOAb).

Their medical history, symptoms at admission, medications, length of hospitalization, thorax computerized tomography (CT) findings, oxygenation, and vital signs were recorded. The results for complete blood counts, alanine aminotransferase (ALT), creatinine, high sensitive C-reactive protein (hs-CRP), lactate dehydrogenase (LDH), ferritin, d-dimer, and procalcitonin values were collected from the laboratory information system. Blood samples that were taken within 48 hours of admission were centrifuged and serum aliquots were stored at −20°C. Serum TSH, FT3, FT4, TGAb, TPOAb, follicle-stimulating hormone (FSH), luteinizing hormone (LH), testosterone, and estradiol levels were measured from these samples. Written consent has been obtained from each patient after full explanation of the purpose and nature of all procedures used.

The patients were classified into six categories: euthyroid, subclinical hypothyroidism, ESS, subclinical hyperthyroidism, overt hyperthyroidism, and central hypothyroidism by two investigators in a double-blinded manner as described in references [14–17] (Table 2). Seventy patients were diagnosed as euthyroid according to the reference range for TSH (0.4–4 mU/L) [14]. Four patients were diagnosed as subclinical hypothyroidism if TSH is 4–10 mU/L and FT3 and FT4 levels were in the normal reference range [14]. ESS was diagnosed if the patient had low/normal TSH, low FT3, and normal/low/high FT4. Patients with ESS were subdivided as mild, moderate, and severe according to their thyroid function tests. Low FT3, normal TSH, and FT4 were classified as ESS associated with mild disease; low FT3, normal/low TSH, and normal/low/high FT4 were classified as ESS associated with moderate disease. Low TSH, FT3, and FT4 were classified as ESS associated with severe disease [15]. Patients with TSH <0.4 mU/L and normal FT3 and FT4 levels were diagnosed as subclinical hyperthyroidism [16]. 9 patients had subclinical hyperthyroidism. Eight patients were diagnosed as overt hyperthyroidism if TSH was <0.4 mU/L and FT4 and/or FT3 were higher than the reference range [17]. Patients were diagnosed as central hypothyroidism if TSH, FT4, and FT3 levels were low together with low FSH and LH levels and low sex hormones.

The severity of COVID-19 patients was classified into 1–10 according to the WHO criteria [18].

### 2.1. Biochemical Analysis

Complete blood counts were measured with Unicel DxH800 Coulter Cell Analyzer (Beckman Coulter, USA) from K2EDTA samples. Serum LDH, creatinine, and ALT parameters were analyzed with AU 680 (Beckman Coulter, USA) spectrophotometrically. Ferritin levels were measured with a two-site immunoenzymatic assay in Access Analyzer (Beckman Coulter, USA). D-dimer parameter was quantitated with an immunoturbidimetric assay in 3.2% sodium citrated venous plasma (STA Compact, Diagnostica Stago, France). hs-CRP levels were measured nephelometrically (BN Prospec, Dade Behring, Germany). TSH, FT3, FT4, TPOAb, and TGAb parameters were measured by paramagnetic particle, chemiluminescent immunoassays in serum samples (DxI800, Beckman Coulter, USA). The reference range for TSH was 0.34–5.60 mU/L, for FT3 was 2.6–4.37 ng/L (0.061–0.103 pmol/L), and for FT4 was 0.61–1.12 ng/dl (0.144–0.26 pmol/L). TGAb was 0–115 IU/ml, and TPOAb was 0–34 IU/ml.

FSH and LH levels were also measured by paramagnetic particle, chemiluminescent immunoassays in serum samples (DxI800, Beckman Coulter, USA). Estradiol and testosterone levels were determined by electrochemiluminescence immunoassay (Modular Analytics E170, Roche Diagnostics, Germany).

### 2.2. Statistical Analysis

The comparison of the continuous variables among independent groups was performed with Mann–Whitney *U* and Kruskal–Wallis tests. Consequent measurements were analyzed with the Wilcoxon test. The cross tables of categorical variables were analyzed with chi-square and Fisher's exact tests. The correlation between numerical variables was tested with Spearman's correlation test. The uni- and multivariate binary logistic regression analyses were performed and odds ratios were reported. L2 (Euclidean) cluster analysis was performed with random start points, where *k* = 2 clusters were created. *p* < 0.05 was considered statistically significant. All analyses were executed by using Stata 15.1 software (Stata Corp, Texas 77845 USA).

## 3. Results

Baseline characteristics of patients are shown in Table 2. Median basal lymphocyte percent of the patients was 18.7% (3.3–51.6%; IQR: 15), ferritin was 165 *μ*g/L (5.3–2770 *μ*g/L; IQR: 351), LDH was 267 U/L (126–979 U/L; IQR: 134.5), CRP was 32.4 mg/L (1–317 mg/L; IQR: 70.7), D-dimer was 0.645 mg/L (0.15–20 mg/L; IQR: 0.75), TSH was 1.16 mU/L (0.08–6.26 mU/L; IQR: 1.29), FT4 was 0.97 mng/dl (0.46–1.84 ng/dl; IQR: 0.27), and FT3 was 2.52 ng/L (0.23–5.3 ng/L; IQR: 0.84). Two patients (0.97%) had TGAb positivity and 11 patients (5.36%) had TPOAb positivity. Within this group, 4 patients were euthyroid, 6 patients had ESS, and 1 patient had hyperthyroidism.

Patients were categorized from 1 to 10 according to the WHO illness severity score. Age (rho = 0.35, *p* < 0.001), length of hospitalization (rho = 0.69, *p* < 0.001), neutrophil count (rho = 0.28, *p* < 0.001), ferritin (rho = 0.27, *p* < 0.001), LDH (rho = 0.29, *p* < 0.001), hs-CRP (rho = 0.39, *p* < 0.001), d-dimer (rho = 0.36, *p* < 0.001), procalcitonin (rho = 0.36, *p* < 0.001), and FT4 (rho = 0.2, *p*=0.004) had weak to moderate positive correlations. However, lymphocyte percent (rho = -0.33, *p* < 0.001), FT3 (rho = -0.34, *p* < 0.001), and TSH (rho = -0.21, *p*=0.002) had weak negative correlations with WHO scores. The median WHO score of noneuthyroid patients (score = 5) was significantly higher than that of euthyroid patients (score=4) (*p* < 0.001). Also, moderate ESS patients had higher WHO score (score = 6) when compared to mild ESS patients (score = 5) (*p* < 0.001).

### 3.1. Comparison of Laboratory Parameters and Outcomes of Euthyroid and Noneuthyroid Patients

Euthyroid patients were younger (*p* < 0.001) and mostly female (*p*=0.002). The symptoms at admission were similar in both groups. None of the euthyroid patients had died. Additionally, the length of hospitalization was shorter (*p* < 0.001), and the rate of oxygen demand (*p* < 0.001), ICU admission (*p*=0.022), and mortality (*p*=0.029) were lower in the euthyroid group. Median ferritin (99.3 *μ*g/L vs. 200 *μ*g/L, *p* < 0.001), LDH (230 U/L vs. 284 U/L, *p*=0.013), hs-CRP (18.2 mg/L vs. 51.5 mg/L, *p* < 0.001), procalcitonin (0.07 *μ*g/L vs. 0.1 *μ*g/L, *p* < 0.001), and d-dimer (0.48 mg/L vs. 0.79 mg/L, *p* < 0.001) levels of the euthyroid group were significantly low, and lymphocyte percent (23.7% vs. 17.3%, *p*=0.001) was significantly high.

### 3.2. Comparison of Laboratory Parameters and Outcomes of Patients with ESS

One hundred and eight patients were categorized as ESS: 57 were mild and 51 were moderate. They had higher levels of neutrophil count, LDH, hs-CRP, ferritin, d-dimer, and procalcitonin and lower levels of lymphocyte percent when compared to euthyroid patients. Moreover, subgroup analysis showed that age, neutrophil, and lymphocyte percent, LDH, CRP, ferritin, d-dimer, and procalcitonin levels were significantly different in moderate ESS in comparison with euthyroid and mild ESS cases (Table 3). As clinical outcomes, the length of hospitalization was longer in the moderate ESS group in comparison with both the euthyroid and mild ESS groups (*p* < 0.001). More patients in the moderate ESS group needed oxygen (*p* < 0.001). The mortality of the patients with ESS was significantly higher than that of the euthyroid patients (*p*=0.043). The ICU demand of moderate ESS patients was significantly higher than that of the euthyroid and mild ESS groups (*p*=0.001).

### 3.3. Characteristics of Patients with Other Thyroid Dysfunctions

Eight patients (3.9%) were diagnosed as overt hyperthyroidism. Five of them had previous thyroid function tests and were euthyroid. Three have needed ICU admission and 1 had died. Nine patients (4.3%) had subclinical hyperthyroidism. Three of them had previous thyroid function tests and they were euthyroid. One has needed ICU admission, and nobody had died. Thirteen patients were categorized as ESS and hyperthyroid (high FT4, low TSH, and FT3 levels). When all hyperthyroid patients were considered (*n* = 30, 14.6%), they were older (*p*=0.007) and have had higher d-dimer levels (*p*=0.002). Four patients (1.95%) had subclinical hypothyroidism. They were antibody negative and 3 of them were normal before COVID-19. They have not needed ICU. Three patients (1.46%) were categorized as central hypothyroidism with low FSH and LH levels. Among them, one patient had died.

### 3.4. Characteristics and Laboratory Parameters of Patients Admitted to ICU and Who Had Died

Thirty-one patients were admitted to ICU. They were older in age (*p* < 0.001) and their length of hospitalization was longer (*p* < 0.001). D-dimer, procalcitonin, hs-CRP, LDH, ferritin, and neutrophil counts were significantly higher in the ICU group together with low lymphocyte percent (Table 4). Nine patients had died. They were also older in age (*p*=0.001), and their length of hospitalization was longer (*p*=0.006). Their neutrophil count, procalcitonin, and d-dimer levels were significantly higher (Table 5).

FT3 and TSH levels were significantly lower in the ICU group (*p* < 0.001 and *p*=0.005, respectively). FT4 levels were higher, but this difference was not significant (*p*=0.12). Also, in patients who died, FT3 (*p*=0.0025) and TSH levels were significantly lower (*p*=0.02).

### 3.5. Predictive Factors of Mortality and ICU Admission

Univariate logistic regression analysis showed that age, lymphocyte percent, hs-CRP concentration, procalcitonin, and FT3 levels had a significant relation with mortality (Table 6). Age, length of hospitalization, respiratory rate, basal neutrophil count, ferritin, CRP, LDH, D-dimer, TSH, FT3, and FT4 had a significant relation with ICU admission (Table 6).

We developed a multivariate model with FT3 and age on prediction of mortality. According to this model, increasing age (odds ratio (OR) = 1.06, 95% CI = 1.007–1.12, *p*=0.027) and decreasing FT3 (OR = 0.27, 95 %CI = 0.085–0.86, *p*=0.027) were associated with increased mortality (*p* < 0.001, pseudo-*R*^2^ = 0.21). For the prediction of ICU admission, we developed a multivariate model with age, basal lymphocyte percent, and TSH levels. Increasing age (OR = 1.05, 95% CI = 1.02–1.09, *p*=0.001), decreasing lymphocyte percent (OR = 0.89, 95% CI = 0.84–0.95, *p* < 0.001), and decreasing TSH (OR = 0.57, 95% CI = 0.34–0.95, *p*=0.032) were associated with increased ICU admission risk (*p* < 0.001, pseudo-*R*^2^ = 0.24).

### 3.6. High- and Low-Risk Cluster Analysis

Two clusters (*k* = 2) were formed by using three variables on thyroid functions with L2 (Euclidean) cluster analysis with random start points. The patients in the cluster with high risk had a mortality ratio of 7.48% (*n* = 8) compared to patients in low-risk cluster (1.11%, *n* = 1) (*p*=0.039).

The patients in high-risk cluster had a lower median FT3 value (median = 2.34 ng/L; IQR = 0.86) compared to patients in low-risk cluster (median = 2.67 ng/L; IQR = 0.71) (*p* < 0.001). The patients in high-risk cluster had a higher median FT4 value (median = 1.04 ng/dL; IQR = 0.33) (*p* < 0.001) compared to patients in low-risk cluster (median = 0.93 ng/dL; IQR = 0.2). The patients in high-risk cluster had a lower median TSH value (median = 0.62 mIU/L; IQR = 0.59) compared to patients in low-risk cluster (median = 1.89 mIU/L; IQR = 1.37) (*p*=0.005).

Regarding mortality, the positive predictive value of the high-risk cluster was 7.48% (95% CI = 3.28%–14.2%) and negative predictive value was 98.9% (95% CI = 94.2%–100%); sensitivity was 88.9% (95% CI: 51.8%–99.7%), and specificity was 48.2% (95% CI: 40.9%–55.5%).

The high-risk group included 8 of the 9 patients who had died (88.8%, *p*=0.039) and 25 of 31 patients admitted to ICU (80.6%; *p*=0.001).

### 3.7. Evaluation of Thyroid Function Tests according to Previous Tests

When previous tests of patients within the last 12 months were evaluated, current median TSH levels were lower than previous levels, but there was not a significant difference (*n* = 90, *p*=0.058). Additionally, current FT3 levels were significantly lower (*n* = 34, *p* < 0.001) while for 69 patients, the current FT4 levels were significantly higher (*p* < 0.001). 32 patients had follow-up TSH levels and there was a statistically significant increase (*p*=0.046), 16 patients had FT3 and there was not a significant increase (*p*=0.055), and 29 patients had FT4 levels and there was a statistically significant decrease (*p*=0.01) according to levels during infection. 10 patients who had euthyroid sick syndrome were euthyroid in control tests. 11 patients who were euthyroid were euthyroid in control tests, 2 patients who were euthyroid were subclinical hypothyroid in follow-up. 2 patients who were hyperthyroid were euthyroid in control tests. From two patients who were subclinical hypothyroid during COVID-19 infection, one was euthyroid and one was subclinical hypothyroid in control tests. One patient who was central hypothyroid during COVID-19 infection was central hypothyroid in the control blood test. When his previous thyroid function status was checked, he was found to be euthyroid.

## 4. Discussion

Thyroid dysfunction rate was 65.8% in this study (21.4% low TSH, 52.6% low FT3, 21.9% high FT4, and 1.95% high TSH). Additionally, we had 108 ESS, 9 subclinical hyperthyroidism, 8 overt thyrotoxicoses, 4 subclinical hypothyroidism, and 3 central hypothyroidism cases. The inflammatory markers, clinical severity score, mortality, and ICU admission were found to be higher in patients with ESS. Thyrotoxicosis was not associated with increased inflammatory markers, mortality, or ICU admission. Thyroid dysfunction was present in all nine patients who had died.

The severe adult respiratory syndrome (SARS) epidemic in 2002 has had multiorgan effects; even thyroid follicular and parafollicular damages were found in autopsy specimens [19]. ACE-2 is known to be the receptor for coronavirus entry and a recent study demonstrated their presence in thyroid cell cultures [20]. Therefore, this might explain the possible direct effect of COVID-19 on the thyroid gland. Muller et al. [5] showed that thyrotoxicosis was mostly evident in COVID-19 + high-intensity ICU (HICU) patients in comparison with patients with COVID-19 + low-intensity ICU (LICU) and COVID-19-HICU. They have revealed the cause of thyrotoxicosis as an atypical form of subacute thyroiditis in which neck pain was absent due to lymphopenia [5]. Two other studies showed thyrotoxicosis and have interpreted the thyroid dysfunction as thyroiditis primarily. They suggested that the underlying mechanism for thyrotoxicosis was either cytokine storm or direct effect of SARS-CoV-2 by ACE-2 receptor [6,9].

We found thyrotoxicosis in 17 patients. Moreover, 13 patients had thyrotoxicosis together with ESS as they had suppressed TSH levels with increased FT4 and decreased FT3 levels, a condition which is hard to make a differential diagnosis. Also, in the studies from Italy, Lania et al. and Muller et al. noted that there might be the coexistence of ESS and thyrotoxicosis in a group of their patients [5, 6]. None of our patients have experienced pain. Only one of these patients was antibody positive, so the possible underlying mechanism might be destructive thyroiditis. Unfortunately, we have not thought to measure thyroglobulin.

Severe COVID-19 was related to a consequent decrease in the Treg/Th17 cell ratio, which might result in aggravated inflammatory responses and organ damage [21]. Viral infections are known to activate autoimmunity also by molecular mimicry mechanisms [22]. Thus, it is obvious that there is a link between the pathogenesis of COVID-19 and autoimmune thyroid disease. In one study, SARS-CoV-2 monoclonal antibodies were applied to different tissues and the thyroid gland was reactive, which has proved that COVID-19 might lead to thyroiditis [23]. However, clinical studies showed contradictory results. In one study, increased TPOAb positivity was detected [24], while increased autoimmunity was not detected in others [9, 12]. In the light of this knowledge, we evaluated the TPOAb and TGAb in 205 patients, but we could not observe an increased rate of antibody positivity according to the normal population [25, 26]. As autoimmunity may develop in later stages, these patients should be followed up regularly.

The most probable mechanism that defines the thyroid dysfunction is ESS. Deiodinases play an important role in the pathogenesis of ESS. In one study, ESS was found to be correlated with disease severity in ICU patients. The possible mechanisms might be the effect of cytokines on HPA axis, thyroid binding proteins, or peripheric metabolism of thyroid hormones [27]. Actually, it is not surprising to think that a severe COVID-19 infection also alters thyroid hormone metabolism and causes ESS. ESS related to COVID-19 was seen in 16–48% of cases in different reports [3, 4, 7–12]. However, these studies have several limitations, such as small sample size [3, 7, 10, 11, 13], drug interferences [3,6], and limited test panel [3, 5, 6, 8]. Only two studies had evaluated thyroid autoimmunity [9, 12], and prior thyroid function tests were assessed only in another [8].

ESS was diagnosed in 16.9% of COVID-19 (*n* = 71) patients with high fatality rate by Zhang et al. [10], and they have correlated thyroid dysfunction to increased neutrophil count, CRP, LDH, and CK, and low lymphocyte count. But this study was retrospective with a relatively small sample size and thyroid autoantibodies were not measured. Campi et al. [12] have declared the ESS rate as 40% of COVID-19 (*n* = 144) patients with higher serum cortisol, CRP, and IL-6 levels. Association of ESS with increased inflammatory markers was also confirmed by Zou et al. [4] who has found FT3 and CRP as predictive factors for disease severity. Gao et al. [7] have evaluated thyroid function tests of 100 severely ill COVID-19 patients and have found reduced FT3 levels associated with all-cause mortality. Shwarz et al. [11] also found low FT3 levels associated with higher mortality and ICU admission. Our ESS rate was 52.6% in 205 patients, making it the second largest study after the study by Lania et al. [6]. We found ESS to be associated with increased inflammatory parameters and WHO severity score, ICU admission, and mortality rates. In contrast to other studies, we have classified ESS patients as mild and moderate. Both inflammatory parameters and disease severity were found to be significantly high in moderate ESS in comparison with the mild ESS and euthyroid patients. In moderate ESS, TSH is normal or low, FT3 is low, and FT4 is high. So it is predicted that low FT3 is not enough for the severity of COVID-19.

In univariate and multivariate regression analysis, low FT3 was found to predict mortality. Also, we created a different scenario with low T3, low TSH, and high T4 by cluster analysis. Accordingly, the patients with a lower median FT3 (*p* < 0.001), higher median FT4 (*p*=0.032), and a lower median TSH values (*p* < 0.001) had significantly higher risk for mortality (*n* = 8; 7.48% vs *n* = 1; 1.11%; *p*=0.039). We also compared these two risk clusters for ICU admission rate, and high risk cluster included 25 of 31 patients admitted to ICU (23.3% vs. 6.67%, *p*=0.001). We believe that risk clusters established for our study according to thyroid functions are valuable for the prediction of ICU admission risk and mortality.

COVID-19-induced thyroid dysfunction might be due to a primary thyroid injury (thyrotoxicosis; atypical thyroiditis), a secondary injury at hypothalamic or pituitary level, or both of them [28]. In this study, three patients were assigned as secondary hypothyroidism confirmed by low FSH and LH levels and low sex hormones. One of these patients had died.

When the strength of our study is considered, it is one of the largest patient groups in the literature with all thyroid function tests, thyroid antibodies, previous thyroid, and pituitary function tests which were evaluated individually in a double-blinded manner by two investigators together with clinical outcomes. But our study also has several limitations. A control group was not enrolled, and follow-up thyroid function tests and antibodies were planned, but patients have refused to come to the hospital because of the pandemic.

There are a lot of conflicts about COVID-19 and thyroid. What we want to say is that if you are a euthyroid, you will be lucky. In this study, the length of hospitalization, the rate of oxygen demand, and ICU admission rate were lower in the euthyroid patients. Moreover, none of the euthyroid patients died. Furthermore, the worst scenario might be to fall into high-risk group. Hence, the prognosis of patients who are in high-risk cluster with low FT3 (median = 2.34 ng/L; IQR = 0.86), a high median FT4 value (median = 1.04 ng/dL; IQR = 0.33), and a low median TSH value (median = 0.62 mIU/L; IQR = 0.59) are poor and mortality is increased.

We believe that COVID-19 will have effects on the thyroid gland, especially in respect to autoimmunity, and the pituitary gland. Future studies with follow-up measurements should be planned.

## Figures and Tables

**Table 1:** Evaluation of clinical studies about thyroid and COVID-19 infection.

Study	Study population	Euthyroid	ESS	Hyperthyroid	Hypothyroid	Thyroid dysfunction (TD) and lab	Conclusion	Limitation
Chen et al. [3]	50 COVID-19/54 controls/50 non-COVID-19 controls	36%	30%	Not determined	Not determined	Not determined	Both ↓TSH and ↓TT3 may be important in the course of COVID-19	Retrospective. Total hormones measured. Drug interference. Pituitary hormones not measured.
Zou et al. [4]	149 patients	72%	27.5%	Not determined	Not determined	ESS associated with ↓lymphocyte, ↑sedimentation, ↑CRP, ↑procalcitonin	Both FT3 and CRP predict COVID-19 severity	Retrospective, small groups, drug interference.
Muller et al. [5]	HICU-2019: 78, HICU-2020: 85, and LICU-2020: 41 patients	Not determined	Not determined	HICU-2020: 15%, HICU-2019: 1%, LICU-2020: 2%	Not determined	Not determined	Atypic thyroiditis was associated with COVID-19	Thyroid hormones not measured in all patients. Thyroid imaging 2 months after infection.
Lania et al. [6]	287 patients	74.6%	Not determined	9.4% scl. 20.2% overt	5.2% scl. 2 overt	Thyrotoxicosis related to ↑IL-6	Thyrotoxicosis may be associated with COVID-19	Drug interference. Thyroid hormone not measured in all patients.
Gao et al. [7]	100 patients	Not determined	28%	Not determined	8%	↓FT3 related to ↑CRP, IL-6, TNF-*α* in survivors	FT3 <3.10 pmol/l had ↑all-cause mortality	Most patients were severely ill.
Khoo et al. [8]	334 COVID-19, 122 control	86.6%	Not determined	5.4%	5.7%	↓TSH related to ↑CRP and ↑cortisol ↑FT4 related to ↑CRP	Most patients euthyroid had mild reduction in TSH and FT4	Single center. Clinical severity not evaluated.
Lui et al. [9]	191 patients	87%	Not determined	7.3%	0.5%	↓T3 related to ↑sedim, CRP, LDH	↓T3 related to COVID-19 severity	No control group. Thyroid hormone not measured in all patients.
Zhang et al. [10]	71 patients	64%	16.9%	5.6%	12.6%	TD related to ↑neutrophil, ↑CRP, ↑LDH, ↑CK, ↓lymphocyte	TD related to ↑fatality rate, ↑length of hospitalization	Retrospective. Small study population. Antibodies were not measured.
Schwarz et al. [11]	54 patients	63%	37%	Thyroid hormones	Not determined	Low T3 related to death, ventilation and ICU	FT3 level can serve as a prognostic marker for disease severity	Small study population.
Campi et al. [12]	115 patients	48%	33%	Not determined	Not determined	↑Cortisol, CRP, IL-6 levels high in patients with ESS	Low T3 related to mortality	No control group
Malik et al. [13]	48 COVID-19, 28 control	21%	Not determined	Not determined	Not determined	IL-6 was associated with abnormal thyroid function tests	TSH and TT3 levels were lower in COVID-19 patients	Small cohort, short follow-up. FT3, FT4, and pituitary hormones were not measured.
This study	205 patients (single center)	34.1%	52.6%	14.6%	3.4%	CRP, d-dimer, ferritin, procalcitonin were high, and lymphocyte was low in ESS	High-risk cluster with a lower median FT3, a higher median FT4 value, and a lower median TSH value included 9 of 11 died patients.	No control group. Autoantibodies were measured in early period.

ESS: euthyroid sick syndrome; HICU: high-intensity intensive care unit; LICU: low-intensity intensive care unit; TNF-*α*: tumor necrosis factor-alpha; IQR: interquartile range; CRP: C-reactive protein; TSH: thyroid-stimulating hormone; LDH: lactate dehydrogenase; FT3: free triiodothyronine; FT4: free thyroxine; scl: subclinical; RT3: reverse triiodothyronine.

**Table 2:** Baseline characteristics of patients.

Parameter	*n*	%
Sex		
Female	92	44.88
Male	113	55,12

Symptoms		
Weakness	144	70.24
Cough	123	60
Shortness of breath	104	50.7
Myalgia	80	39
Fever	76	37

Comorbidities		
Hypertension	87	42.6
Type 2 DM	54	26.3
Coronary artery disease	31	15.2
Chronic obstructive pulmonary disease	25	12.3
Malignancy	12	5.85
Cerebrovascular disease	7	3.43

Mortality	9	4.39

Intensive care unit admission	31	15.12

CT findings	178	88.56

Oxygen demand	132	64.3
Nasal prongs (*n*, %)	56	27.3
NIMV or reservoir mask (*n*)	46	22.4
IMV (*n*)	30	14.6

Thyroid function status		
Euthyroid sick syndrome	108	52.6
Mild	57	27.8
Moderate	51	24.8
Euthyroid	70	34.1
Subclinical hyperthyroidism	9	4.3
Hyperthyroidism	8	3.9
Subclinical hypothyroidism	4	1.95
Central hypothyroidism	3	1.46

NIMV: noninvasive mechanical ventilation; IMV: invasive mechanical ventilation; DM: diabetes mellitus.

**Table 3:** Baseline characteristics and laboratory parameters of euthyroid patients with ESS and its subgroups.

Parameter	*n*	Median (min-max)	IQR	*p* value
Age				<0.001
Euthyroid	70	51 (21–87)	17	
ESS	108	62.5 (26–94)	22	
Euthyroid	70	51 (21–87)	17	
Mild ESS	57	61 (26–94)	22	
Moderate ESS	51	65 (31–90	22	<0.001

Sex (F/M)				0.004
Euthyroid	(42/28)	—	—	
ESS	(41/67)	—	—	
Euthyroid	(42/28)	—	—	
Mild ESS	(24/33)	—	—	
Moderate ESS	(17/34)	—	—	0.01

Lymphocyte percent				
Euthyroid	70	23.7 (3.5–45.7)	15.7	<0.001
ESS	108	16.95 (3.3–46.7)	11.75	
Euthyroid	70	23.7 (3.5–45.7)	15.7	
Mild ESS	57	19.3 (3.3–46.7)	11.2	
Moderate ESS	51	12.9 (3.3–39.1)	11.1	<0.001

Neutrophil count ×10^3^/*μ*L)				
Euthyroid	70	3.45 (1–9.5)	2.4	0.02
ESS	108	3.85 (0.9–21.3)	2.65	
Euthyroid	70	3.45 (1–9.5)	2.4	
Mild ESS	57	3.6 (0.9–14.7)	2.4	
Moderate ESS	51	3.9 (1.5–21.3)	2.5	0.018

Thrombocyte count 10^3^/*μ*L)				0.07
Euthyroid	70	195 (72.6–387)	70	
ESS	108	169 (36–525)	85	
Euthyroid	70	195 (72.6–387)	70	
Mild ESS	57	169 (37–499)	75	0.13
Moderate ESS	51	180 (36–562)	106	

Ferritin (*μ*g/L)				<0.001
Euthyroid	68	99.3 (5.2–1295)	199	
ESS	107	205 (14–2770)	392	
Euthyroid	68	99.3 (5.2–1295)	199	
Mild ESS	57	167 (14–2770)	304	
Moderate ESS	51	294 (34–2338)	457	<0.001

LDH (U/L)				0.01
Euthyroid	69	230 (126–728)	102	
ESS	108	280 (131–979)	152	
Euthyroid	69	230 (126–728)	102	
Mild ESS	57	261 (143–979)	94	
Moderate ESS	51	308 (131–831)	217	0.01

hs-CRP (mg/L)				
Euthyroid	70	18.2 (2–179)	45.3	0.001
ESS	108	52.1 (1–317)	81.7	
Euthyroid	70	18.2 (2–179)	45.3	
Mild ESS	57	36 (1–223)	66.2	<0.001
Moderate ESS	51	66.2 (3.9–317)	105	

Procalcitonin (*μ*g/L)				<0.001
Euthyroid	65	0.07 (0.02–0.98)	0.05	
ESS	108	0.1 (0.02–20.5)	0.09	
Euthyroid	65	0.07 (0.02–0.98)	0.05	
Mild ESS	57	0.08 (0.02–0.73)	0.07	
Moderate ESS	51	0.12 (0.04–20.5)	0.38	<0.001

D-dimer (mg/L)				<0.001
Euthyroid	69	0.48 (0.27–20)	0.48	
ESS	108	0.85 (0.15–18)	1.1	
Euthyroid	69	0.48 (0.27–20)	0.48	
Mild ESS	57	0.53 (0.15–11.3)	0.58	
Moderate ESS	51	1.26 (0.23–18)	1.09	<0.001

ESS: euthyroid sick syndrome; LDH: lactate dehydrogenase; CRP: C-reactive protein; IQR: interquartile range.

**Table 4:** Characteristics and laboratory parameters of patients admitted to ICU.

Parameter	*n*	Median (min-max)	IQR	*p* value
Age				<0.001
Admitted to ICU	31	68 (42–90)	18	
Not admitted to ICU	174	55.5 (21–94)	18	

Length of hospitalization				<0.001
Admitted to ICU	31	26 (7–116)	28	
Not admitted to ICU	174	9 (2–40)	7	

Lymphocyte percent				
Admitted to ICU	31	12.5 (3.3–39.1)	8.5	
Not admitted to ICU	174	20.55 (3.6–51.6)	15.5	<0.001

Neutrophil count (×10^3^/*μ*l)				
Admitted to ICU	31	5.2 (1.4–21.3)	5.2	
Not admitted to ICU	174	3.5 (0.9–18.2)	2.2	<0.001

Thrombocyte count (×10^3^/*μ*L)				
Admitted to ICU	31	171 (89–562)	128	

Not admitted to ICU	174	186.5 (36–562)	81	0.92

Ferritin (*μ*g/L)				
Admitted to ICU	31	241 (11–2770)	634	
Not admitted to ICU	168	147.8 (5.3–2338)	315.6	0.02

LDH (U/L)				
Admitted to ICU	31	320 (131–744)	207	
Not admitted to ICU	173	257 (126–979)	122	<0.001

CRP (mg/L)				
Admitted to ICU	31	75.3 (4.58–317)	82.7	
Not admitted to ICU	174	25.5 (1–289)	65	<0.001

Procalcitonin (*μ*g/L)				
Admitted to ICU	31	0.13 (0.3–20.5)	0.06	
Not admitted to ICU	169	0.07 (0.02–2.47)	0.07	<0.001

D-dimer (mg/L)				
Admitted to ICU	31	1.58 (0.38–20)	1.59	
Not admitted to ICU	173	0.59 (0.15–3.84)	0.55	<0.001

TSH (mU/L)				
Admitted to ICU	31	0.82 (0.08–2.46)	0.77	
Not admitted to ICU	174	1.25 (0.09–6.26)	1.38	0.005

FT4 (ng/dL)				
Admitted to ICU	31	1.11 (0.61–1.67)	0.49	
Not admitted to ICU	170	0.97(0.46–1.84)	0.26	0.12

FT3 (ng/L)				
Admitted to ICU	31	2.06 (1.01–5.3)	1.03	
Not admitted to ICU	169	2.58 (0.23–4.49)	0.7	<0.001

ICU: intensive care unit; LDH: lactate dehydrogenase; CRP: C-reactive protein; LDH: lactate dehydrogenase; TSH: thyroid-stimulating hormone; FT4: free thyroxine; FT3: free triiodothyronine; IQR: interquartile range.

**Table 5:** Characteristics and laboratory parameters of patients who had died and survived from COVID-19.

Parameter	*n*	Median (min-max)	IQR	*p* value
Age				
Survived	196	58 (21–94)	18.5	
Exitus	9	76 (57–87)	13	0.001

Length of hospitalization				
Survived	196	10.5 (2–116)	9	
Exitus	9	21 (7–51)	11	0.006

Lymphocyte percent				
Survived	196	19.2 (3.3–51.6)	15.8	
Exitus	9	12.3 (3.3–17.1)	4.9	0.005

Neutrophil count (×10^3^/*μ*l)				
Survived	196	3.6 (0.9–21.3)	2.55	
Exitus	9	5.2 (3.1–11)	44	0.004

Thrombocyte count (×10^3^/*μ*L)				
Survived	196	182 (36–562)	82	
Exitus	9	195 (130–327)	128	0.39

Ferritin (*μ*g/L)				
Survived	190	166 (5.3–2770)	351	
Exitus	9	147 (34.4–985)	303	0.9

LDH (U/L)				
Survived	195	261 (126–979)	137	
Exitus	9	300 (165–589)	83	0.11

hs-CRP (mg/L)				
Survived	196	31.8 (1–289)	70.5	
Exitus	9	61.1 (12.7–317)	176	0.056

Procalcitonin (*μ*g/L)				
Survived	191	0.08 (0.02–2.47)	0.08	
Exitus	9	0.13 (0.07–20.5)	0.03	0.02

D-dimer (mg/L)				
Survived	195	0.63 (0.15–20)	0.74	
Exitus	9	1.68 (0.6–7.15)	1.49	0.0053

TSH (mU/L)				
Survived	196	1.22 (0.09–6.26)	1.27	
Exitus	9	0.5 (0.08–2.46)	0.85	0.02

FT4 (ng/dL)				
Survived	192	0.97 (0.46–1.84)	0.26	
Exitus	9	1.04 (0.61–1.51)	0.64	0.82

FT3 (ng/L)				
Survived	191	2.57 (0.23–5.3)	0.81	
Exitus	9	1.69 (1.01–3.3)	1.03	0.0025

LDH: lactate dehydrogenase; CRP: C-reactive protein; LDH: lactate dehydrogenase; TSH: thyroid-stimulating hormone; FT4: free thyroxine; FT3: free triiodothyronine; IQR: interquartile range.

**Table 6:** Univariate logistic regression analysis of parameters associated with mortality and ICU admission.

	Odds ratio (%)	Standard error	95% CI	*p* value	Pseudo-*R*^2^
Age (years)					
Mortality	1.08	0.031	1.02–1.15	<0.001	0.14
ICU	1.06	0.016	1.02–1.09	<0.001	0.1

Length of hospitalization (days)					
Mortality	1.02	0.015	1.0002–1.05	0.07	0.04
ICU	1.17	0.032	1.1–1.23	<0.001	0.38

Lymphocyte percent					
Mortality	0.87	0.048	0.78–0.97	0.003	0.11
ICU	0.89	0.02	0.84–0.94	<0.001	0.12

D-dimer (mg/L)					
Mortality	1.077	0.087	0.91–1.26	0.42	0.0086
ICU	2.36	0.55	1.48–3.75	<0.001	0.18

hs-CRP (mg/L)					
Mortality	1.01	0.004	1.001–1.018	0.01	0.07
ICU	1.007	0.027	1.0–1.012	0.009	0.03

FT3 (ng/L)					
Mortality	0.209	0.107	0.07–0.57	0.001	0.13
ICU	0.44	0.13	0.24–0.804	0.005	0.04

TSH (mU/L)					
Mortality	0.36	0.21	0.11–1.14	0.03	0.06
ICU	0.46	0.13	0.26–0.805	0.001	0.06

FT4 (ng/dL)					
Mortality	0.92	1.32	0.055–15.3	0.95	0.00
ICU	4.6	3.47	1.05–20.1	0.04	0.02

Ferritin (*μ*g/L)					
Mortality	1	0.001	0.99–1	0.9	0.0002
ICU	1.00	0.0003	1.00–1.001	0.03	0.02

Procalcitonin (*μ*g/L)					
Mortality	1.37	0.35	0.83–2.27	0.01	0.08
ICU	1.33	0.42	0.71–2.4	0.052	0.02

ICU: intensive care unit; LDH: lactate dehydrogenase; CRP: C-reactive protein; LDH: lactate dehydrogenase; TSH: thyroid-stimulating hormone; FT4: free thyroxine; FT3: free triiodothyronine; CI: confidence interval.

## Data Availability

Data used to support the findings of this study are available from the corresponding author upon request.
